# CONSORT extension for the reporting of randomised controlled trials conducted using cohorts and routinely collected data (CONSORT-ROUTINE): checklist with explanation and elaboration

**DOI:** 10.1136/bmj.n857

**Published:** 2021-04-30

**Authors:** Linda Kwakkenbos, Mahrukh Imran, Stephen J McCall, Kimberly A McCord, Ole Fröbert, Lars G Hemkens, Merrick Zwarenstein, Clare Relton, Danielle B Rice, Sinéad M Langan, Eric I Benchimol, Lehana Thabane, Marion K Campbell, Margaret Sampson, David Erlinge, Helena M Verkooijen, David Moher, Isabelle Boutron, Philippe Ravaud, Jon Nicholl, Rudolf Uher, Maureen Sauvé, John Fletcher, David Torgerson, Chris Gale, Edmund Juszczak, Brett D Thombs

**Affiliations:** 1 Behavioural Science Institute, Clinical Psychology, Radboud University, Nijmegen, Netherlands; 2 Lady Davis Institute for Medical Research, Jewish General Hospital, Montreal, Canada; 3 National Perinatal Epidemiology Unit Clinical Trials Unit, Nuffield Department of Population Health, University of Oxford, Oxford, UK; 4 Center for Research on Population and Health, Faculty of Health Sciences, American University of Beirut, Ras Beirut, Lebanon; 5 Basel Institute for Clinical Epidemiology and Biostatistics, Department of Clinical Research, University Hospital Basel, University of Basel, Basel, Switzerland; 6 Örebro University, Faculty of Health, Department of Cardiology, Örebro, Sweden; 7 Meta-Research Innovation Center at Stanford (METRICS), Stanford University, Palo Alto, USA; 8 Meta-Research Innovation Centre Berlin (METRIC-B), Berlin Institute of Health, Berlin, Germany; 9 Department of Family Medicine, Western University, London, Canada; 10 ICES, Toronto, Canada; 11 Centre for Clinical Trials and Methodology, Barts Institute of Population Health Science, Queen Mary University, London, UK; 12 Department of Psychology, McGill University, Montréal, Québec, Canada; 13 Faculty of Epidemiology and Population Health, London School of Hygiene and Tropical Medicine, London, UK; 14 Department of Paediatrics, University of Toronto, Toronto, Canada; 15 Division of Gastroenterology, Hepatology, and Nutrition and Child Health Evaluative Sciences, SickKids Research Institute, The Hospital for Sick Children, Toronto, Canada; 16 Department of Health Research Methods, Evidence, and Impact, McMaster University, Hamilton, Canada; 17 Health Services Research Unit, University of Aberdeen, Aberdeen, UK; 18 Library Services, Children's Hospital of Eastern Ontario, Ottawa, Canada; 19 Department of Cardiology, Clinical Sciences, Lund University, Lund, Sweden; 20 University Medical Centre Utrecht, Utrecht, Netherlands; 21 University of Utrecht, Utrecht, Netherlands; 22 Centre for Journalology, Clinical Epidemiology Program, Ottawa Hospital Research Institute, Ottawa, Canada; 23 Université de Paris, Centre of Research Epidemiology and Statistics (CRESS), Inserm, INRA, Paris, France; 24 Centre d’Épidémiologie Clinique, Assistance Publique–Hôpitaux de Paris (AP-HP), Hôpital Hôtel Dieu, Paris, France; 25 School of Health and Related Research, University of Sheffield, Sheffield, UK; 26 Department of Psychiatry, Dalhousie University, Halifax, Canada; 27 Scleroderma Society of Ontario, Hamilton, Canada; 28 Scleroderma Canada, Hamilton, Canada; 29 *The BMJ*; 30 York Trials Unit, Department of Health Sciences, University of York, York, UK; 31 Neonatal Medicine, School of Public Health, Faculty of Medicine, Imperial College London, Chelsea and Westminster campus, London, UK; 32 Nottingham Clinical Trials Unit, University of Nottingham, University Park, Nottingham, UK; 33 Departments of Psychiatry; Epidemiology, Biostatistics, and Occupational Health; Medicine; and Educational and Counselling Psychology; and Biomedical Ethics Unit, McGill University, Montreal, Canada

## Abstract

Randomised controlled trials are increasingly conducted as embedded, nested, or using cohorts or routinely collected data, including registries, electronic health records, and administrative databases, to assess if participants are eligible for the trial and to facilitate recruitment, to deliver an embedded intervention, to collect trial outcome data, or a combination of these purposes. This report presents the Consolidated Standards of Reporting Trials (CONSORT) extension for randomised controlled trials conducted using cohorts and routinely collected data (CONSORT-ROUTINE). The extension was developed to look at the unique characteristics of trials conducted with these types of data with the goal of improving reporting quality in the long term by setting standards early in the process of uptake of these trial designs. The extension was developed with a sequential approach, including a Delphi survey, a consensus meeting, and piloting of the checklist. The checklist was informed by the CONSORT 2010 statement and two reporting guidelines for observational studies, the Strengthening the Reporting of Observational Studies in Epidemiology (STROBE) statement and the REporting of studies Conducted using Observational Routinely collected Data (RECORD) statement. The extension includes eight items modified from the CONSORT 2010 statement and five new items. Reporting items with explanations and examples are provided, including key aspects of trials conducted using cohorts or routinely collected data that require specific reporting considerations.

Well designed and conducted randomised controlled trials are the so called gold standard of healthcare intervention research.^1^ ^2^ ^3^ The use of reporting guidelines, including the Consolidated Standards of Reporting Trials (CONSORT) statement, improves the transparency and completeness of publications of the results of randomised controlled trials.^4^ ^5^ ^6^ ^7^ The CONSORT 2010 statement facilitates critical appraisal and interpretation of randomised controlled trials by providing guidance to authors on a minimum set of items that should be reported for all trials.^8^ ^9^ The aim of the CONSORT 2010 statement was to improve the reporting of two-arm parallel group randomised controlled trials. Extensions of the CONSORT statement have been developed to encourage better reporting of other trial designs, including, for example, multi-arm parallel group randomised trials,^10^ cluster trials,^11^ pilot and feasibility trials,^12^ and pragmatic trials.^13^

Interest in randomised controlled trials conducted using cohorts^14^ or with routinely collected data is growing. Routinely collected data includes registries,^15^ ^16^ electronic health records,^17^ and administrative databases, such as government or private health insurance databases, social care databases, or education databases.^18^ In a cohort, a group of individuals is collected for the purpose of conducting research^14^ whereas routinely collected data refer to data initially collected for purposes other than research or without specific a priori research questions developed before collection.^19^ ^20^ Trials might use a cohort or routinely collected data to identify eligible participants, to determine outcomes, to implement an intervention, or for a combination of these purposes. For example, in registry based randomised controlled trials, a registry could be used to identify eligible participants for a trial, for the collection of baseline characteristics of the participants, and as the source of outcome data; some registries have used interactive technology to actively flag participants to enrol in randomised controlled trials when patient data are entered into the registry.^16^ In some trials involving electronic health records, the electronic health record itself is used to implement an intervention. For example, one randomised controlled trial tested an intervention to reduce prescribing of antibiotics by feeding back personalised antibiotic prescription data to primary care physicians.^21^ Trials that use a cohort or routinely collected data to identify and recruit participants and to collect outcome data might be referred to as embedded or nested, whereas others might use the cohort or routinely collected data for one purpose or the other.

The use of cohorts and routinely collected data might make randomised controlled trials easier and more feasible to perform by reducing cost, time, and other resources, and could facilitate the conduct of trials that more closely replicate real world clinical practice by supporting recruitment of large and representative samples.^22^ ^23^ These trial designs, however, are relatively recent innovations, and published randomised controlled trial reports might not describe important aspects of their methodology in a standardised way. Trials conducted using cohorts and routinely collected data share certain elements with conventional randomised controlled trials, but distinctive elements to report also exist, which are not covered in the CONSORT 2010 statement (box 1).^8^ ^9^ Because of the substantial overlap in the design, conduct, analysis, and reporting of trials conducted in cohorts and with different types of routinely collected data, we developed one CONSORT extension for the reporting of randomised controlled trials conducted using cohorts and routinely collected data (CONSORT-ROUTINE).

Box 1 **Key methodological issues and considerations in trials conducted using cohorts and routinely collected data**

**Design**

- Trials conducted using cohorts or routinely collected databases might differ from conventional trial designs because they use these sources of data to identify eligible participants; automate randomisation; deliver an intervention; collect data, including assessing outcomes; or a combination of these functions.
- Some trials might use a hybrid approach that integrates the use of these sources of data and trial specific methods for functions such as delivery of the intervention and assessing outcomes.
- Cohorts and routinely collected databases can vary substantially in the way they represent complete, random, or convenience samples. Because the cohort or routinely collected database could serve as the sampling frame for the trial, the representativeness of participants in the trial might depend on the characteristics of the database.
- The comprehensiveness, collection procedures, and type of demographic or outcome data available in a cohort or routinely collected database could influence the design of the trial, including the research question, eligibility criteria of the trial, and the choice of outcomes.
- The timing between identifying eligibility, delivering the intervention, and assessing the outcomes might be governed by the frequency of data collection in a cohort or routinely collected database, and is less controllable by trial investigators than in conventional trials.
- In trials using cohorts or routinely collected data, informed consent could be applied at different levels and in different ways compared with conventional trial designs. Consent might be sought and obtained to use the cohort or routinely collected database and for the trial, and consent that would typically be expected to occur in conventional trials might not be done because of features of the integrated cohort or database and trial design.

**Conduct**

- Because cohorts, registries, electronic health records, and administrative databases vary in the way they are set up for research, clinical care, or financial and administrative purposes, the completeness and accuracy of the data might vary substantially between different databases and between variables within one database.
- Challenges could arise in linking routinely collected data to other sources of data, including linkage errors when records cannot be linked or are linked incorrectly.

**Analysis**

- A unique feature of trials using cohorts and routinely collected data is that investigators can often access information on participants not enrolled in the trial. Differences in baseline characteristics of eligible people from the cohort or routinely collected database who do not participate in the trial can often be compared with trial participants to inform judgments on the representativeness of the participants in the trial and the generalisability of the results.

**Interpretation**

- Potential differences between the trial target population, people included in the cohort or routinely collected database, and participants in the trial, can influence the applicability of the trial results and should be considered when interpreting the findings.
- Limitations to the use of a cohort or routinely collected data for a trial include constraints on available outcome measures and issues with data linkage, data validation, and data quality that could influence eligibility for the trial and assessment of outcomes.

**Summary points**

- Trials might use a cohort or routinely collected data to identify eligible participants, to determine outcomes, to implement an intervention, or for a combination of these purposes.
- These trial designs are relatively recent innovations, and published randomised controlled trial reports might not describe important aspects of their methodology in a standardised way.
- A CONSORT extension was developed for the reporting of randomised controlled trials conducted using cohorts and routinely collected data (CONSORT-ROUTINE)

## Development and scope of the CONSORT extension

The project was registered with the Enhancing the QUAlity and Transparency Of health Research (EQUATOR) Network,^24^ and a protocol was published.^23^ The extension was developed after a consensus driven process^25^ and included: confirmation of the need for a reporting guideline; a scoping review to assess reporting quality and identify reporting considerations to include in a preliminary checklist version^26^; a three round Delphi process to collect input on the checklist items from stakeholders, including reporting guideline developers, funders, journal editors, patient representatives, trial methodologists, epidemiologists, meta-research authors, ethicists, biostatisticians, and clinical trialists who were identified by members of the project team; a consensus meeting to advise on items to include and the checklist structure; and publication, dissemination, and implementation of the final checklist. Details on methods and results from each stage of the process are described elsewhere.^27^ In brief, 27 items for consideration were initially developed by members of the CONSORT Extension Project Team based on review of items included in the CONSORT 2010 statement,^8^ ^9^ the Strengthening the Reporting of Observational Studies in Epidemiology (STROBE)^28^ statement, and the REporting of studies Conducted using Observational Routinely collected Data (RECORD)^29^ statement, and also discussions with steering committee members. All items were rated in Delphi round 1. In Delphi round 2, 13 items were rated, and in round 3, 11 items were rated. Response rates for the Delphi study were 92 of 125 (74%) invited participants in round 1, 77 of 92 (84%) participants who completed round 1 in round 2, and 62 of 77 (81%) participants who completed round 2 in round 3. Members of the project team attended an in-person consensus meeting where the Delphi results were considered and a preliminary checklist was developed. The preliminary version of the checklist was pilot tested by 17 people with experience in trials conducted using cohorts or routinely collected data. In all stages of development, key stakeholders in trials research and potential end users of the CONSORT extension were involved, including participants from a wide range of scientific disciplines and with diverse experience in conducting trials in cohorts and with different types of routinely collected databases.

Consistent with other CONSORT statements, this extension describes a minimum set of information that should be reported and provides a checklist to facilitate compliance. The extension applies to randomised controlled trials conducted using one or more cohorts or routinely collected databases to: identify, recruit, or consent eligible participants; implement an intervention; collect trial data, including outcomes; or a combination of these purposes. For randomised controlled trials that use cohorts or routinely collected data to only assess outcomes, some extension items might not be relevant.

The extension includes eight items from the CONSORT 2010 statement that were modified and five new items. No items were removed from the CONSORT 2010 checklist. Table 1 shows the extension items compared with the CONSORT 2010 checklist. Table 2 is the integrated extension checklist. Box 2 summarises important changes to the CONSORT 2010 statement.

**Table 1:** Checklist for reporting of trials conducted using cohorts or routinely collected data: comparison of the extension with the CONSORT 2010 statement

Section/topic	Item No	CONSORT 2010 checklist item	Extension for trials conducted using cohorts or routinely collected data
**Title and abstract**			
	1a	Identification as a randomised trial in the title	
	1b	Structured summary of trial design, methods, results, and conclusions (for specific guidance see CONSORT for abstracts)	Structured summary of trial design, methods, results, and conclusions (for specific guidance see CONSORT for abstracts). Specify that a cohort or routinely collected data were used to conduct the trial and, if applicable, provide the name of the cohort or routinely collected database(s) (modified)
**Introduction**			
Background and objectives	2a	Scientific background and explanation of rationale	—
	2b	Specific objectives or hypotheses	—
**Methods**			
Trial design	3a	Description of trial design (such as parallel, factorial) including allocation ratio	Description of trial design (such as parallel, factorial) including allocation ratio, that a cohort or routinely collected database(s) was used to conduct the trial (such as electronic health record, registry) and how the data were used within the trial (such as identification of eligible trial participants, trial outcomes) (modified)
	3b	Important changes to methods after trial commencement (such as eligibility criteria), with reasons	—
Cohort or routinely collected database (new section heading)	ROUTINE-1	—	Name, if applicable, and description of the cohort or routinely collected database(s) used to conduct the trial, including information on the setting (such as primary care), locations, and dates (such as periods of recruitment, follow-up, and data collection) (new)
	ROUTINE-2	—	Eligibility criteria for participants in the cohort or routinely collected database(s) (new)
	ROUTINE-3		State whether the study included person-level, institutional-level, or other data linkage across two or more databases and, if so, linkage techniques and methods used to evaluate completeness and accuracy of linkage (new)
Trial participants (modified from “Participants”)	4a	Eligibility criteria for participants	Eligibility criteria for trial participants, including information on how to access the list of codes and algorithms used to identify eligible participants, information on accuracy and completeness of data used to ascertain eligibility, and methods used to validate accuracy and completeness (eg, monitoring, adjudication), if applicable (modified)
	4b	Settings and locations where the data were collected	—
	ROUTINE-4	—	Describe whether and how consent was obtained (new)
Interventions	5	The interventions for each group with sufficient details to allow replication, including how and when they were actually administered	—
Outcomes	6a	Completely defined pre-specified primary and secondary outcome measures, including how and when they were assessed	Completely defined pre-specified primary and secondary outcome measures, including how and when they were ascertained and the cohort or routinely collected database(s) used to ascertain each outcome (modified)
	ROUTINE-5	—	Information on how to access the list of codes and algorithms used to define or derive the outcomes from the cohort or routinely collected database(s) used to conduct the trial, information on accuracy and completeness of outcome variables, and methods used to validate accuracy and completeness (eg, monitoring, adjudication), if applicable (new)
	6b	Any changes to trial outcomes after the trial commenced, with reasons	—
Sample size	7a	How sample size was determined	—
	7b	When applicable, explanation of any interim analyses and stopping guidelines	—
Sequence generation	8a	Method used to generate the random allocation sequence	—
	8b	Type of randomisation; details of any restriction (such as blocking and block size)	—
Allocation concealment mechanism	9	Mechanism used to implement the random allocation sequence (such as sequentially numbered containers), describing any steps taken to conceal the sequence until interventions were assigned	Mechanism used to implement the random allocation sequence (such as embedding an automated randomiser within the cohort or routinely collected database(s)), describing any steps taken to conceal the sequence until interventions were assigned (modified)
Implementation	10	Who generated the random allocation sequence, who enrolled participants, and who assigned participants to interventions	—
Blinding	11a	If done, who was blinded after assignment to interventions (for example, participants, care providers, those assessing outcomes) and how	—
	11b	If relevant, description of the similarity of interventions	—
Statistical methods	12a	Statistical methods used to compare groups for primary and secondary outcomes	—
	12b	Methods for additional analyses, such as subgroup analyses and adjusted analyses	—
**Results**			
Participant flow (diagram is strongly recommended)	13a	For each group, the numbers of participants who were randomly assigned, received intended treatment, and were analysed for the primary outcome	For each group, the number of participants in the cohort or routinely collected database(s) used to conduct the trial and the numbers screened for eligibility, randomly assigned, offered and accepted interventions (eg, cohort multiple RCTs), received intended treatment, and analysed for the primary outcome (modified)
	13b	For each group, losses and exclusions after randomisation, together with reasons	—
Recruitment	14a	Dates defining the periods of recruitment and follow-up	—
	14b	Why the trial ended or was stopped	—
Baseline data	15	A table showing baseline demographic and clinical characteristics for each group	—
Numbers analysed	16	For each group, number of participants (denominator) included in each analysis and whether the analysis was by original assigned groups	—
Outcomes and estimation	17a	For each primary and secondary outcome, results for each group, and the estimated effect size and its precision (such as 95% confidence interval)	—
	17b	For binary outcomes, presentation of both absolute and relative effect sizes is recommended	—
Ancillary analyses	18	Results of any other analyses performed, including subgroup analyses and adjusted analyses, distinguishing pre-specified from exploratory	—
Harms	19	All important harms or unintended effects in each group (for specific guidance see CONSORT for harms)	—
**Discussion**			
Limitations	20	Trial limitations, addressing sources of potential bias, imprecision, and, if relevant, multiplicity of analyses	—
Generalisability	21	Generalisability (external validity, applicability) of the trial findings	—
Interpretation	22	Interpretation consistent with results, balancing benefits and harms, and considering other relevant evidence	Interpretation consistent with results, balancing benefits and harms, and considering other relevant evidence, including the implications of using data that were not collected to answer the trial research questions (modified)
**Other information**			
Registration	23	Registration number and name of trial registry	—
Protocol	24	Where the full trial protocol can be accessed, if available	—
Funding	25	Sources of funding and other support (such as supply of drugs), role of funders	Sources of funding and other support for both the trial and the cohort or routinely collected database(s), role of funders (modified)

RCT=randomised controlled trial.

**Table 2:** Combined CONSORT 2010 and CONSORT-ROUTINE checklist

Section/topic	Item No	CONSORT extension for trials conducted using cohorts or routinely collected data item	Reported on page No
**Title and abstract**			
	1a	Identification as a randomised trial in the title	
	1b	Structured summary of trial design, methods, results, and conclusions (for specific guidance see CONSORT for abstracts). Specify that a cohort or routinely collected data were used to conduct the trial and, if applicable, provide the name of the cohort or routinely collected database(s)	
**Introduction**			
Background and objectives	2a	Scientific background and explanation of rationale	
	2b	Specific objectives or hypotheses	
**Methods**			
Trial design	3a	Description of trial design (such as parallel, factorial) including allocation ratio, that a cohort or routinely collected database(s) was used to conduct the trial (such as electronic health record, registry) and how the data were used within the trial (such as identification of eligible trial participants, trial outcomes)	
	3b	Important changes to methods after trial commencement (such as eligibility criteria), with reasons	
Cohort or routinely collected database	ROUTINE-1	Name, if applicable, and description of the cohort or routinely collected database(s) used to conduct the trial, including information on the setting (such as primary care), locations, and dates (such as periods of recruitment, follow-up, and data collection)	
	ROUTINE-2	Eligibility criteria for participants in the cohort or routinely collected database(s)	
	ROUTINE-3	State whether the study included person-level, institutional-level, or other data linkage across two or more databases and, if so, linkage techniques and methods used to evaluate completeness and accuracy of linkage	
Trial participants	4a	Eligibility criteria for trial participants, including information on how to access the list of codes and algorithms used to identify eligible participants, information on accuracy and completeness of data used to ascertain eligibility, and methods used to validate accuracy and completeness (eg, monitoring, adjudication), if applicable	
	4b	Settings and locations where the data were collected	
	ROUTINE-4	Describe whether and how consent was obtained	
Interventions	5	The interventions for each group with sufficient details to allow replication, including how and when they were actually administered	
Outcomes	6a	Completely defined pre-specified primary and secondary outcome measures, including how and when they were ascertained and the cohort or routinely collected database(s) used to ascertain each outcome	
	ROUTINE-5	Information on how to access the list of codes and algorithms used to define or derive the outcomes from the cohort or routinely collected database(s) used to conduct the trial, information on accuracy and completeness of outcome variables, and methods used to validate accuracy and completeness (eg, monitoring, adjudication), if applicable	
	6b	Any changes to trial outcomes after the trial commenced, with reasons	
Sample size	7a	How sample size was determined	
	7b	When applicable, explanation of any interim analyses and stopping guidelines	
Sequence generation	8a	Method used to generate the random allocation sequence	
	8b	Type of randomisation; details of any restriction (such as blocking and block size)	
Allocation concealment mechanism	9	Mechanism used to implement the random allocation sequence (such as embedding an automated randomiser within the cohort or routinely collected database(s)), describing any steps taken to conceal the sequence until interventions were assigned	
Implementation	10	Who generated the random allocation sequence, who enrolled participants, and who assigned participants to interventions	
Blinding	11a	If done, who was blinded after assignment to interventions (for example, participants, care providers, those assessing outcomes) and how	
	11b	If relevant, description of the similarity of interventions	
Statistical methods	12a	Statistical methods used to compare groups for primary and secondary outcomes	
	12b	Methods for additional analyses, such as subgroup analyses and adjusted analyses	
**Results**			
Participant flow (a diagram is strongly recommended)	13a	For each group, the number of participants in the cohort or routinely collected database(s) used to conduct the trial and the numbers screened for eligibility, randomly assigned, offered and accepted interventions (eg, cohort multiple RCTs), received intended treatment, and analysed for the primary outcome	
	13b	For each group, losses and exclusions after randomisation, together with reasons	
Recruitment	14a	Dates defining the periods of recruitment and follow-up	
	14b	Why the trial ended or was stopped	
Baseline data	15	A table showing baseline demographic and clinical characteristics for each group	
Numbers analysed	16	For each group, number of participants (denominator) included in each analysis and whether the analysis was by original assigned groups	
Outcomes and estimation	17a	For each primary and secondary outcome, results for each group, and the estimated effect size and its precision (such as 95% confidence interval)	
	17b	For binary outcomes, presentation of both absolute and relative effect sizes is recommended	
Ancillary analyses	18	Results of any other analyses performed, including subgroup analyses and adjusted analyses, distinguishing pre-specified from exploratory	
Harms	19	All important harms or unintended effects in each group (for specific guidance see CONSORT for harms)	
**Discussion**			
Limitations	20	Trial limitations, addressing sources of potential bias, imprecision, and, if relevant, multiplicity of analyses	
Generalisability	21	Generalisability (external validity, applicability) of the trial findings	
Interpretation	22	Interpretation consistent with results, balancing benefits and harms, and considering other relevant evidence, including the implications of using data that were not collected to answer the trial research questions	
**Other information**			
Registration	23	Registration number and name of trial registry	
Protocol	24	Where the full trial protocol can be accessed, if available	
Funding	25	Sources of funding and other support for both the trial and the cohort or routinely collected database(s), role of funders	

RCT=randomised controlled trial.

This checklist is also presented separately in web table 1.

Box 2 **Summary of major changes to the CONSORT 2010 statement**

**New items—introduces five new items that are specific to randomised controlled trials conducted using cohorts or routinely collected data**

- ROUTINE-1 on the description of the cohort or routinely collected database(s)
- ROUTINE-2 on the eligibility criteria for participants in the cohort or routinely collected database(s)
- ROUTINE-3 on data linkage across two or more databases
- ROUTINE-4 on consent for use of cohort or routinely collected data and trial participation
- ROUTINE-5 on codes and algorithms used to define or derive the outcomes from the cohort or routinely collected database(s)

**Modified items—modifies eight CONSORT 2010 items**

- Item 1b on specifying that a cohort or routinely collected data were used in the abstract
- Item 3a on specifying that a cohort or routinely collected data were used in the trial design
- Item 4a on eligibility criteria for trial participants
- Item 6a on outcome measures
- Item 9 on implementation of the random allocation sequence
- Item 13a on the participant flow
- Item 22 on the interpretation of results
- Item 25 on the sources of funding

For each modified and new item, this document describes the item, identifies whether the item was modified or new, provides examples of good reporting, explains the rationale for including the item, and elaborates on reporting considerations. For items that were not modified from the CONSORT 2010 statement, but for which reporting considerations exist for trials conducted using cohorts or routinely collected data, we have also provided an example and explanation. Examples of good reporting were retrieved from primary and secondary trial reports and, in some cases, trial protocols. For all items, the explanations provided supplement those in the CONSORT 2010 explanation and elaboration statement.^8^ ^9^ Adequate reporting of many trials conducted using cohorts or routinely collected data will also require reference to other CONSORT extensions (www.consort-statement.org/extensions), including those for cluster trials,^11^ pragmatic trials,^13^ and others. The CONSORT-ROUTINE explanation and elaboration statement only deals with reporting issues relevant to the use of cohorts and routinely collected data in trial design and conduct, and readers should consult other relevant extensions.

## Title and abstract

### 

#### Item 1a (unmodified)

Identification as a randomised trial in the title.

#### Examples

“Bivalirudin versus heparin in non-ST and ST-segment elevation myocardial infarction-a registry-based randomized clinical trial in the SWEDEHEART registry (the VALIDATE-SWEDEHEART trial).”^30^

“Clinical effectiveness and cost-effectiveness of a multifaceted podiatry intervention for falls prevention in older people: a multicentre cohort randomised controlled trial (the REducing Falls with ORthoses and a Multifaceted podiatry intervention trial).”^31^

#### Explanation

Item 1a is meant to aid in indexing and identifying randomised controlled trial reports in electronic databases. The title, at a minimum, should contain recognisable terminology identifying the study as a randomised trial. If word count permits, the type of trial (eg, cohort multiple randomised controlled trials, registry based randomised controlled trials) or the cohort or routinely collected database(s) used to conduct the trial (eg, SWEDEHEART registry) should be provided.

#### Item 1b (modified)

CONSORT 2010 item: Structured summary of trial design, methods, results, and conclusions (for specific guidance see CONSORT for abstracts).

Modified CONSORT extension item: Structured summary of trial design, methods, results, and conclusions (for specific guidance see CONSORT for abstracts). Specify that a cohort or routinely collected data were used to conduct the trial and, if applicable, provide the name of the cohort or routinely collected database(s).

#### Examples

“The TIMING study is a national, investigator-led, registry-based, multicentre, open-label, randomised controlled study. The Swedish Stroke Register is used for enrolment, randomisation and follow-up.”^32^

“The Department of Veterans Affairs (VA) MI-Plus study was a cluster-randomized trial involving 168 community-based primary care clinics and 847 providers in 26 states, the Virgin Islands, and Puerto Rico . . . with the clinic as the randomization unit. We collected administrative data for 15,847 post-MI [myocardial infarction] patients and medical record data for 10,452 of these.”^33^

#### Explanation

Abstracts are used for electronic database indexing and are the most commonly read sections of articles.^9^ ^34^ They provide information on the trial methodology and main results, and allow readers to evaluate if the study likely covers their information needs. In addition to CONSORT 2010 abstract elements, abstracts of trials using cohorts or routinely collected databases should clearly describe the type of cohort or routinely collected database used (eg, registry based trial), according to the examples above. The name of the cohort or database(s) used should also be reported, if applicable. Some databases, such as electronic health records, are typically unnamed, in which case stating that an electronic health record was used is enough. Ideally, the abstract will clarify the purpose for which the cohort or routinely collected database was used (eg, to identify eligible participants, to assess outcomes). More information related to the use of cohorts or routinely collected data that should be reported might also exist, depending on the specific trial design. Whenever possible, authors should report their abstract in a structured format.^8^ ^9^

## Introduction

### Background and objectives

#### Item 2a (unmodified)

Scientific background and explanation of rationale (see CONSORT 2010).^8^ ^9^

#### Item 2b (unmodified)

Specific objectives or hypotheses (see CONSORT 2010).^8^ ^9^

## Methods

### Trial design

#### Item 3a (modified)

CONSORT 2010 item: Description of trial design (such as parallel, factorial) including allocation ratio.

Modified CONSORT extension item: Description of trial design (such as parallel, factorial) including allocation ratio, that a cohort or routinely collected database(s) was used to conduct the trial (such as electronic health record, registry) and how the data were used within the trial (such as identification of eligible trial participants, trial outcomes).

#### Examples

“The Determination of the Role of Oxygen in Suspected Acute Myocardial Infarction (DETO2X-AMI) trial was a multicenter, parallel-group, open-label, registry-based, randomized, controlled trial in which routine supplemental oxygen therapy was compared with ambient air in the treatment of patients with suspected myocardial infarction who did not have hypoxemia at baseline. The trial used the national comprehensive Swedish Web System for Enhancement and Development of Evidence-Based Care in Heart Disease Evaluated According to Recommended Therapies (SWEDEHEART) . . . for patient enrollment and data collection.”^35^

“PATIENT was a parallel arm, pragmatic clinical trial in which 21,752 adults were randomized to receive either UC [usual care] or 1 of 2 interventions designed to increase adherence to statins, angiotensin-converting enzyme inhibitors (ACEIs), and angiotensin receptor blockers (ARBs) . . . Using each region’s EMR [electronic medical record], we identified participants 40 years and older with diabetes mellitus and/or cardiovascular disease (CVD), suboptimally (<90%) adherent to a statin or ACEI/ARB during the previous 12 months, and due or overdue for a refill . . . Within each region, we randomly assigned a sample of eligible members to the 3 primary study arms (usual care and 2 intervention arms) in a 1:1:1 ratio at the study outset . . . We used the EMR to capture age, race, gender, healthcare utilization for diabetes and CVD, and BP [blood pressure] and lipid levels.”^36^

#### Explanation

According to CONSORT 2010, authors should describe the trial design (eg, parallel group, cluster randomised), conceptual framework (eg, superiority, equivalence, or non-inferiority), and allocation ratio (eg, 1:1, 2:1). Also, they should describe that one or more cohorts or routinely collected databases were used, how they were used (eg, to identify eligible participants, to deliver the intervention, to collect data including to assess outcomes), and whether their use influenced other methodological choices that might have implications for how the results of the trial are interpreted and apply to different populations. Examples include constraints on the eligibility criteria for the trial; timing between evaluating eligibility, delivery of the intervention, and assessing outcomes; and outcomes available.

#### Item 3b (unmodified)

Important changes to methods after trial commencement (such as eligibility criteria), with reasons (see CONSORT 2010).^8^ ^9^

Trial methods and procedures might depend on protocols (eg, eligibility criteria, outcomes assessed of cohorts or routinely collected databases). Changes to protocols that affect aspects of trial methods, such as identification of eligible participants, outcome variables collected, or timing of outcome assessments, should be described (see also ROUTINE-1 and ROUTINE-2).

### Cohort or routinely collected database (new section subheading)

#### Item ROUTINE-1 (new)

Name, if applicable, and description of the cohort or routinely collected database(s) used to conduct the trial, including information on the setting (such as primary care), locations, and dates (such as periods of recruitment, follow-up, and data collection).

#### Examples

“Family practices in England, Scotland, or Wales were eligible for the study if they were contributing data to the Clinical Practice Research Datalink (CPRD). The CPRD is a large database that includes the EHRs [electronic health records] of ≈ 7% of all UK general practices from 1987 to the present.”^37^

“The [Scleroderma Patient-centered Intervention Network] SPIN Cohort is a convenience sample. Eligible SPIN Cohort patients are recruited at SPIN sites (https://www.spinsclero.com/en/sites) during regular medical visits, and written informed consent is obtained. A medical data form is submitted online by the site to enrol participants. Cohort participants complete outcome measures via the internet upon enrolment and subsequently every 3 months. SPIN Cohort enrollment started in March 2014 and is ongoing.”^38^

#### Explanation

This new section covers a wider description of the cohort or routinely collected database that is different from the description of how the cohort or database was used in the trial, which is covered in section 4 (trial participants). Providing the name of the cohort or routinely collected database allows readers to identify other studies, including trials, conducted with the same cohort or database and consider if the results apply to their setting. A description of the cohort or routinely collected database, including geographical locations and clinical settings, enables readers to assess characteristics relevant to understanding the sampling frame for recruitment of participants to the trial and the potential validity of the data for the research question. The authors should provide references to any publications that have described the cohort or database methods, or characteristics of the included participants. A rationale for why the specific cohort or routinely collected database was used for the trial should be provided.

Characteristics that could influence data quality should be reported and, if applicable, include the reason for data collection (eg, clinical care, administrative purposes), and the time period and related procedures by which data are collected, among others. Information on surgical procedures, for example, might be complete and accurate for administrative data derived from physician billing because reimbursement depends on its accuracy. Associated diagnostic codes, however, might be less reliable if these codes are not essential for billing. For data collected with electronic health records in the UK, for example, data that relate to items detailed in the Quality Outcomes Framework are likely of better quality if captured after 2004.^39^ Any changes in cohort or routinely collected database procedures, such as frequency of data collection or items collected, could lead to changes in outcome variables in randomised controlled trials or other aspects of trial conduct and should be reported.

#### Item ROUTINE-2 (new)

Eligibility criteria for participants in the cohort or routinely collected database(s).

#### Examples

“Patients were eligible for inclusion in the cohort if they were 45 years or older; had a smoking history of at least 10 pack-years; had a clinical diagnosis of mild-to-severe COPD [chronic obstructive pulmonary disease], defined as a postbronchodilator forced expiratory volume in 1s (FEV1) to forced vital capacity ratio of 0.7 or lower and a postbronchodilator FEV1 of at least 30%, according to Global Initiative of Chronic Obstructive Lung Disease (GOLD) and American Thoracic Society and European Respiratory Society criteria (GOLD stage 1–3); and had at least one documented or self-reported exacerbation during the past 3 years, with the restriction that the last exacerbation had ended at least 4 weeks before inclusion and symptoms had returned to patients’ baseline levels. Exclusion criteria were poor mastery of the Dutch language, poor cognitive functioning, known allergy to doxycycline, pregnancy, and a life expectancy of shorter than 1 month.”^40^

“Baseline characteristics and clinical outcomes will be extracted from routinely recorded clinical data held in the NNRD [National Neonatal Research Database]. The NNRD holds data from all infants admitted to National Health Service (NHS) neonatal units in England, Scotland and Wales (~90 000 infants annually). Contributing neonatal units are known as the UK Neonatal Collaborative. Data are extracted from point-of-care neonatal electronic health records completed by health professionals during routine clinical care. A defined data extract, the Neonatal Dataset of ~450 data items, is transmitted quarterly to the Neonatal Data Analysis Unit at Imperial College London and Chelsea and Westminster NHS Foundation Trust where patient episodes across different hospitals are linked and data are cleaned (queries about discrepancies and implausible data configurations are fed back to health professionals and rectified).”^41^

#### Explanation

Because the cohort or routinely collected database serves as the sampling frame for the trial, the representativeness of the participants in the trial depends on the eligibility criteria, and a clear description of criteria for entry into the cohort or routinely collected database should be provided.^29^ For example, in health administrative data, having insurance (eg, Medicare in the United States) is a prerequisite for having a record in the database; a randomised controlled trial with participants recruited from the database could only be representative of people with insurance coverage.

When a cohort or routinely collected database in which eligibility fluctuates over time is used (eg, health insurance data), researchers should clearly specify how eligibility was defined and how changes in eligibility over the study period were managed. Also, changes in variable coding over time could result in differences in characteristics of participants considered eligible for enrolment in the randomised controlled trial. Therefore, coding changes relevant to characterising participants in the cohort or database, and eligibility criteria and enrolment in the randomised controlled trial, should be reported.

#### Item ROUTINE-3 (new)

State whether the study included person-level, institutional-level, or other data linkage across two or more databases and, if so, linkage techniques and methods used to evaluate completeness and accuracy of linkage.

#### Examples

[Information in main text] “Individuals on the Oregon Experiment “reservation list” (N=100 407) were probabilistically matched to individual OCHIN [Oregon Community Health Information Network] patients (N=106 692), using Link Plus software and demographic variables common to both data sets. Two researchers independently performed a case-by-case review of uncertain matches using additional demographic variables. Appendix Table 1 provides more details.”

[Information in appendix] “To identify individuals common to both the Medicaid reservation list and the OCHIN patient population, we used Link Plus software to probabilistically compare demographic variables contained in both datasets. Matching variables included first and last name, date of birth, gender, street address, city, Oregon Medicaid identification number, and preferred language. The software generates a “match score” indicating each pair’s likelihood of being a match. For pairs of uncertain match status based on match score, we conducted double clerical review by independent reviewers. We also completed several rounds of quality assurance analyses to verify the validity of our match results.”^42^

#### Explanation

When databases are linked, investigators need to select a set of variables to use for linking, determine the best method for linking the databases and develop a linking algorithm, and evaluate the accuracy of linkages between the databases.^43^ A description of linkage methods and the success of linkage is critical to allow the reader to assess the likelihood and potential effect of any linkage error and the possibility of related bias.^44^ Linkage bias occurs when associations are present between the probability of linkage error (eg, false and missing matches) and variables of interest. For example, linkage rates might vary by patient characteristics, such as health status or health services received. Even small errors in the linkage process can introduce bias and lead to results that can overestimate or underestimate the associations being studied.^45^

Authors should describe if linkage of records across multiple databases was conducted and, if so, the methods of linkage (eg, deterministic *v* probabilistic, quality and type of variables used for linkage), how linkage validation was done, and the results of linkage validation with estimated rates of successful linkage. Details should be provided on blocking variables (variables used to form pairs for comparison only among those with the potential to be matches, such as the first three digits of a postal code), completeness of linkage variables, linkage rules, thresholds, and manual review of potential matches, if undertaken.^46^ ^47^ If linkage was conducted before the trial for previous studies or general use, or if linkage was undertaken by an external provider, such as a data linkage centre, a reference describing the data resource and linkage methods should be provided. Authors should report linkage error with standard approaches including comparisons with gold standards or reference datasets, sensitivity analyses, and comparing characteristics of linked and unlinked data.^48^

### Trial participants (modified section subheading)

#### Item 4a (modified)

CONSORT 2010 item: Eligibility criteria for participants.

Modified CONSORT extension item: Eligibility criteria for trial participants, including information on how to access the list of codes and algorithms used to identify eligible participants, information on accuracy and completeness of data used to ascertain eligibility, and methods used to validate accuracy and completeness (eg, monitoring, adjudication), if applicable.

#### Examples

“Primary care physicians were eligible for the study if they practiced in a study clinic, provided care to at least 10 adults with type 2 diabetes, and provided written informed consent to participate. Patients were classified as having diabetes if they had 2 or more out-patient diabetes International Classification of Diseases, Ninth Revision (ICD-9) codes (250.xx) or used 1 or more diabetes-specific medications in the 1-year period before randomization. This diabetes identification method has estimated sensitivity of 0.91 and positive predictive value of 0.94.”^49^

“An EHR [electronic health record]-based algorithm to identify eligible patients was constructed based on International Classification of Diseases, 9th and 10th Revisions, Clinical Modification codes (67–69) (see Table E1 in the online supplement) that are present on admission. In addition, nurses complete a five-item electronic checklist during intake to denote the disease-specific eligibility criteria. To validate the algorithm, we reviewed 271 medical charts across the participating hospitals. The algorithm identified 171 of these patients as eligible and 100 as ineligible. Using manual chart review as the gold standard, the algorithm had a false-positive rate of 1% and a false-negative rate of 5%.”^50^

#### Explanation

This section relates to entry into the trial (rather than the cohort or routinely collected database, which is covered in items ROUTINE-1 to ROUTINE-3). When eligible trial participants were identified from records in a cohort or routinely collected database, authors should report information necessary to evaluate or replicate this process. This information should include a clear and detailed description of all codes, algorithms, and free text field entries, or combinations of these, including any statistical code if possible. Ideally, a link to all material needed for replication should be provided in an appendix or posting to an accessible website.

Use of routinely collected data could introduce some degree of misclassification bias, and information on the validity of participant classification must be specifically described, including reference to available validation studies and any methods used to directly assess the validity of the data used for classification of participants and the accuracy of the classification. Potential changes that could affect different settings and time points should be considered (eg, when coding standards or strategies that might affect the validity of the data are changed, or when software or algorithms are updated).

To help readers assess the applicability of trial results, authors should clearly describe potential differences between the trial target population, people included in the cohort or health database, and actual participants in the trial. Filtering effects could occur, for instance, when data are more often incomplete in special situations, such as emergency visits (compared with routine visits) as a result of different processes for routine data collection, and if people with incomplete data are not screened for trial eligibility.

#### Item 4b (unmodified)

Settings and locations where the data were collected.

#### Examples

“The trial was conducted in the area of the Lille-Douai Health Insurance district (Northern France) during the institutional seasonal influenza vaccination campaign of 2014–2015 . . . In the intervention group, 25 GPs received and were supposed to expose in their waiting rooms 135 pamphlets and one poster (added to the usual mandatory information) withdrawing all the other posters. In the control group, waiting rooms were kept in their usual state . . . Data were extracted between October 15, 2014 and February 28, 2015 from the SIAM-ERASME claim database of the Lille-Douai district Health Insurance Fund on patient level.”^51^

“The present study is one of three trials that took place in the context of the PRO-AGE (PRevention in Older people-Assessment in GEneralists’ practices) project in three locations. The present study was conducted in Hamburg, Germany, and was intended to test whether HRA-O [health risk appraisal for older persons], combined with personal reinforcement and supplemented . . . In Hamburg, general practitioners (GPs) registered in the entire metropolitan area (~500 GPs) were informed via the newsletter of their regional GP association (BDA-Landesverband Hamburg) . . . Survival, nursing home admission, and need for ambulatory nursing care as well as change of residence data were obtained from the GP records and completed with participant and proxy information. At year 1, the HRA-O questionnaire was used for collecting outcome information from all study participants. It was sent to surviving persons in combination with a short questionnaire on self-efficacy in the patient–physician interaction.”^52^

#### Explanation

Information on the settings and locations where the trial was conducted is key to judge the applicability and generalisability of the trial.^8^ ^9^ In trials conducted in cohorts or with routinely collected data, authors should describe where the trial was implemented and specify if differences existed between centres where overall cohort or database data were collected (see item ROUTINE-2) and those involved in the trial. This situation might occur if only a subset of centres in the cohort or database are selected randomly or by characteristics, such as the quality of the data, location, delivery of healthcare, or language. Also, centres in a cohort, for instance, could be assigned to participate in different ongoing trials occurring simultaneously or in overlapping time periods with the same cohort.

#### Item ROUTINE-4 (new)

Describe whether and how consent was obtained.

#### Example

“At enrollment in the cohort, patients are asked to provide informed consent for prospective collection of clinical, survival and PROMs [patient-reported outcome measures] data . . . we ask patients’ consent to be randomly selected to receive offers on experimental interventions in the future and to use their data comparatively . . . Patients within the cohort who meet the inclusion criteria form a subcohort of eligible patients . . . From among this subcohort, a random sample is selected . . . Randomly selected patients are offered the experimental intervention (boost prior to sCRT [standardised chemoradiation therapy]) by their treating physician. If they accept the offer, they will sign an additional informed consent to receive the boost. Patients who refuse the boost will receive care as usual (that is, sCRT). Patients in the subcohort who will not be randomly selected will not be informed about the boost intervention, nor will they be informed about their participation in the control arm of this study.”^53^

#### Explanation

In trials in cohorts and with routinely collected data, informed consent might be applied at different levels and at multiple stages for an individual participant, and in different ways than in conventional randomised controlled trial designs where consent is usually obtained once for treatment, randomisation, and data use.^54^ Reporting the information provided to potential participants and the consent sought will help readers understand what participants knew and what they expected or hoped might happen at each stage of the research, including the trial. Clearly describing this information in the text and in flow diagrams will allow readers to evaluate the applicability of the trial results and facilitate replication.

Authors should describe the different types of consent sought and obtained for the cohort or routinely collected database, and the trial. These might include: consent for use of health data for research from a cohort or routinely collected database; consent to be contacted for future research purposes; prior consent to future randomisation without explicit notice, which often occurs in trials that use the cohort multiple randomised controlled trial design^14^ ^55^; consent to receive a trial intervention; or conventional consent for participation in the trial and randomisation. Other types of consent could also be relevant, such as consent to no description of the experimental intervention if allocated to the control, or consent for linkage with other datasets. For each type of consent sought, authors should describe from whom consent was sought, whether consent was sought for all participants in the trial or only some (eg, only those allocated to a trial intervention), and when each type of consent was sought.

### Interventions

#### Item 5 (unmodified)

The interventions for each group with sufficient details to allow replication, including how and when they were actually given.

#### Example

“We developed a computer-based electronic alert system for identifying consecutive hospitalized OAC [oral-anticoagulation]-naïve patients with AF [arterial fibrillation] and tested the hypothesis that such an alert system would improve OAC prescription. The alert system automatically identified hospitalized patients with AF without an active OAC prescription in the electronic order entry system. The alert system was incorporated into the electronic medical chart and order entry system of the University Hospital in Bern, Switzerland. It recognized AF by permanently searching diagnosis lists and physician notes of the entire electronic patient chart database for free text entries of AF or its various abbreviations. Alerts were issued 24 hours after the onset of hospital stay if . . . 4 criteria for an individual patient were present . . . Once the criteria were fulfilled, the alert was issued in the electronic patient chart. The alert was visible to physicians and nurses, but only physicians were enabled to respond to the alert.”^56^

“Intervention included a single real-time notification by letter to the patient and by electronic message within the KPSC [Kaiser Permanente Southern California] electronic medical record system to each patient’s primary care provider and asthma specialist (if the patient had previously seen one). The patient letters and physician messages noted excessive SABA [short-acting β2-agonist] dispensing, suggestions for management, and facilitated allergy referral recommendation for those patients without prior asthma specialist care . . . Controls received KPSC standard asthma care management without research contact.”^57^

#### Explanation

Interventions are sometimes delivered by electronic health record systems or with an administrative database. Examples provided here describe a clinical decision support tool^56^ and a drug alert system^57^ embedded within electronic health records. Other examples could include reminders or links to a clinical practice guideline when specific disease codes or other patient characteristics (eg, age, sex) that indicate guideline relevance are entered into an electronic health record. Authors should report interventions triggered or delivered by an electronic health record, registry, or administrative database in enough detail so that readers can understand the characteristics of the intervention, replicate the intervention in other research, and implement the intervention clinically. The Template for Intervention Description and Replication (TIDieR) provides guidance for reporting of interventions.^58^

### Outcomes

#### Item 6a (modified)

CONSORT 2010 item: Completely defined pre-specified primary and secondary outcome measures, including how and when they were assessed.

Modified CONSORT extension item: Completely defined pre-specified primary and secondary outcome measures, including how and when they were ascertained and the cohort or routinely collected database(s) used to ascertain each outcome.

#### Examples

“A hard CVD [cardiovascular disease] event, the primary outcome, was defined as the occurrence of any of the following events in the medical record or Medicare/Medicaid data between IMPACT [Improving Mood-Promoting Access to Collaborative Treatment] enrollment date and December 31, 2008: a) fatal MI [myocardial infarction] (International Classification of Diseases, 10th Revision codes I21-I22 the first-listed cause of death), b) laboratory evidence of acute MI (creatine kinase-myocardial band isoenzyme value 93.0 ng/ml or troponin value 90.3 K g/l), c) acute MI diagnosis (ICD-9 code 410), d) fatal stroke (International Classification of Diseases, 10th Revision codes I60-I64 the first-listed cause of death), or e) hemorrhagic (ICD-9 codes 430Y432) or nonhemorrhagic (ICD-9 codes 433.01, 433.11, 433.21, 433.31, 433.91, 434.01, 434.11, and 434.91) stroke diagnosis. Secondary outcomes were fatal/nonfatal MI (categories a-c), fatal/nonfatal MI-cardiac enzyme confirmed (categories a and b), fatal/nonfatal stroke (categories d and e), and all-cause mortality. Death dates were extracted from the Medicare data, and causes of death were obtained from death certificates provided by the Indiana State Department of Health . . . Patients were followed up for a maximum of 7.5 to 9.5 years (median = 8.1 years); however, for cause of death (categories a and d), patients were followed up for a maximum of 5.5 to 7.5 years (median = 6.2 years).”^59^

“The trial used the national comprehensive Swedish Web System for Enhancement and Development of Evidence-Based Care in Heart Disease Evaluated According to Recommended Therapies (SWEDEHEART) . . . for patient enrollment and data collection . . . The primary end point was death from any cause within 365 days after randomization, assessed in the intention-to-treat population. Secondary end points included death from any cause within 30 days after randomization, rehospitalization with myocardial infarction, rehospitalization with heart failure, and cardiovascular death . . . as well as composites of these end points, assessed at 30 days and 365 days . . . Data on the end points of rehospitalization with heart failure and cardiovascular death are not available from SWEDEHEART and must be obtained from the Swedish National Inpatient and Outpatient Registries. Mortality data were obtained from the Swedish National Population Registry, which includes the vital status of all Swedish citizens. All other variables were obtained from SWEDEHEART, which is monitored on a regular basis. Diagnoses at discharge are listed according to codes from the International Classification of Diseases, 10th Revision (ICD-10). The end of follow-up was December 30, 2016, which was 365 days after the last patient underwent randomization. To allow for any lag in registry reporting, the final database was extracted from SWEDEHEART on February 28, 2017, including data on any linked deaths that occurred through December 30, 2016, and reported in the population registry as of February 14, 2017 . . . No central adjudication or trial-specific patient follow-up was performed.”^35^

#### Explanation

All primary and secondary outcomes should be identified and defined, including how and when they were measured, and the cohort(s) or routinely collected database(s) used to ascertain the outcome. The use of routinely collected data might introduce some degree of misclassification. Details on the accuracy and validity of outcome data (eg, classification of participants) must be described, including reference to available validation studies and any methods used to directly assess the validity of data used as primary or secondary outcomes and the accuracy of the data collected. If different databases are used in some sites in the trial, authors should note if outcomes are determined consistently across trial sites.

Because follow-up periods might be considerably longer than recruitment periods, sometimes lasting decades, special attention should be given to potential changes that occur over time that might affect the collection, quality, and completeness of the data. Authors could consider using flow diagrams or special tables to describe these circumstances. A crucial aspect to consider and carefully report is any connection between collection of outcomes and trial arms (eg, detection bias). For example, a comparison of surgery versus non-surgical care should consider that special diagnostic procedures that are routinely done in surgical follow-up visits might not be done in the control group.

#### Item ROUTINE-5 (new)

Information on how to access the list of codes and algorithms used to define or derive the outcomes from the cohort or routinely collected database(s) used to conduct the trial, information on accuracy and completeness of outcome variables, and methods used to validate accuracy and completeness (eg, monitoring, adjudication), if applicable.

#### Example—information on how to access list of codes and algorithms used to define or derive outcomes from cohort or routinely collected database(s) used to conduct the trial

“The primary outcomes were whether or not the patient received preventive care services in the post-period: screenings for cervical, breast, and colorectal cancer (fecal occult blood testing and colonoscopy); screenings for diabetes (glucose and hemoglobin A1c [HbA1c]), hypertension, obesity, and smoking; lipid screening; chlamydia testing; and receipt of influenza vaccination. Codes were used based on EHR [electronic health record] Meaningful Use Stage 1 measures. These included ICD-9-CM [International Classification of Diseases, ninth revision, clinical modification] diagnosis and procedure codes, Current Procedural Terminology and Healthcare Common Procedure Coding System codes, Logical Observation Identifiers Names and Codes, and medication codes. The authors also used relevant code groupings and codes specific to the OCHIN [Oregon Community Health Information Network] EHR, used for Meaningful Use reporting and internal quality improvement initiatives. Appendix Table 2 provides detailed technical specifications and patient eligibility criteria for each measure (see https://ars.els-cdn.com/content/image/1-s2.0-S0749379715004237-mmc1.pdf).”^42^

#### Examples—information on accuracy and completeness of outcome variables, and methods used to validate accuracy and completeness (eg, monitoring, adjudication)

[In supplement]“Uppsala Clinical Research Center provides manuals, education and technical advice, including a telephone help desk for all users of the registry. The system has error checking routines for range and consistency. Definitions are easily available when data are entered. To ensure the correctness of the data entered a monitor visits about 20 hospitals each year and compares data entered into the SWEDEHEART [Swedish Web System for Enhancement and Development of Evidence-Based Care in Heart Disease Evaluated According to Recommended Therapies] with the information in the patients’ records from 30–40 randomly chosen patients in each hospital. When 637 randomly chosen computer forms from 21 hospitals containing 38 121 variables were reviewed in 2007, there was a 96.1% (range: 92.6%-97.4%) agreement.”^60^

“If a patient was suspected to have had a clinical end-point event (i.e., death, myocardial infarction, bleeding, or stroke), the patient’s health care records were subjected to central blinded adjudication to determine the cause of the event according to prespecified criteria.”^61^

#### Explanation

Trials using cohorts or routinely collected data might require specific codes or algorithms, such as diagnostic codes, to identify and define outcomes. An electronic health record query can be performed, for example, with a list of diagnostic codes to identify all patients who have experienced a specific adverse event. An algorithm, or sequence of steps necessary to score or grade an outcome, could also be used. To assess validity and to facilitate reproducibility, the list of codes and algorithms should be provided or linked to an external source within the text or in supplementary material, ideally with the computer code used to reproduce this step.

Cohorts and routinely collected data are often collected and entered by staff involved in routine patient care or by non-clinical staff, based on medical records, and the level of completeness varies. Also, procedures for entering data for clinical care or billing might introduce certain biases, and concerns about data completeness and accuracy could arise.^62^ Authors should describe data completeness in enough detail so that others can evaluate accuracy. Issues of misclassification, and any efforts to minimise misclassification, should be reported.

Outcome definitions might vary between cohorts and routinely collected data, and standards commonly used in clinical trials and data fields might be missing. The authors should describe any adjudication of outcomes, if adjudication was blinded to trial allocation, and which outcome definitions were used (eg, by referring to a separate adjudication protocol).

#### Item 6b (unmodified)

Any changes to trial outcomes after the trial commenced, with reasons (see CONSORT 2010).^8^ ^9^

### Sample size

#### Item 7a (unmodified)

How sample size was determined (see CONSORT 2010).^8^ ^9^

#### Item 7b (unmodified)

When applicable, explanation of any interim analyses and stopping guidelines (see CONSORT 2010).^8^ ^9^

### Randomisation

#### Item 8a (unmodified)

Method used to generate the random allocation sequence (see CONSORT 2010).^8^ ^9^

#### Item 8b (unmodified)

Type of randomisation; details of any restrictions (such as blocking and block size; see CONSORT 2010).^8^ ^9^

### Allocation concealment mechanism

#### Item 9 (modified)

CONSORT 2010 item: Mechanism used to implement the random allocation sequence (such as sequentially numbered containers), describing any steps taken to conceal the sequence until interventions were assigned.

Modified CONSORT extension item: Mechanism used to implement the random allocation sequence (such as embedding the random allocation sequence within the cohort or routinely collected database(s)), describing any steps taken to conceal the sequence until interventions were assigned.

#### Examples

“The [WithHolding Enteral feeds Around packed red cell Transfusion] WHEAT trial is a randomised controlled, unblinded, multicentre, pilot trial comparing two care pathways . . . Infants will be randomised with a 1:1 allocation ratio (using permuted blocks of variable size), stratified within neonatal unit by gestational age at birth and infant sex. Trial processes will be embedded within neonatal EPR [electronic patient record] systems and all outcome data will be extracted from data that are routinely recorded within the existing neonatal EPR systems (BadgerNet and BadgerEPR), and held in the NNRD [National Neonatal Research Database] . . . Infants will be randomised using an online secure central randomisation system which will be embedded into the existing neonatal EPR systems (BadgerNet and BadgerEPR). Randomisation will occur within the EPR to ensure allocation concealment.”^41^

“Randomization to be offered versus not offered, the SPIN-HAND [Scleroderma Patient-centered Intervention Network hand exercise program] intervention will occur at the time of Cohort participants’ regular SPIN Cohort assessments. Eligible Cohort participants, based on questionnaire responses, will be randomized automatically as they complete their regular SPIN Cohort assessments using a feature in the SPIN Cohort platform, which provides immediate centralized randomization and, thus, complete allocation sequence concealment.”^38^

#### Explanation

The use of cohorts or routinely collected data to conduct trials might provide opportunities to embed automated randomisation or selection and allocation algorithms into the cohort or database system to allocate participants to trial arms. This process could be automated or software embedded within the system could communicate with an external randomisation system. If such processes are used, authors should provide enough details for readers to understand the randomisation and allocation concealment processes and to assess how they could influence internal validity.

### Implementation

#### Item 10 (unmodified)

Who generated the random allocation sequence, who enrolled participants, and who assigned participants to interventions (see CONSORT 2010).^8^ ^9^

### Blinding

#### Item 11a (unmodified)

If done, who was blinded after assignment to interventions (eg, participants, care providers, those assessing outcomes) and how (see CONSORT 2010).^8^ ^9^

#### Item 11b (unmodified)

If relevant, description of the similarity of interventions (see CONSORT 2010).^8^ ^9^

### Statistical methods

#### Item 12a (unmodified)

Statistical methods to compare groups for primary and secondary outcomes (see CONSORT 2010).^8^ ^9^

#### Item 12b (unmodified)

Methods for additional analyses, such as subgroup analyses and adjusted analyses (see CONSORT 2010).^8^ ^9^

## Results

### Participant flow (a diagram is strongly recommended)

#### Item 13a (modified)

CONSORT 2010 item: For each group, the numbers of participants who were randomly assigned, received intended treatment, and were analysed for the primary outcome.

Modified CONSORT extension item: For each group, the number of participants in the cohort or routinely collected database(s) used to conduct the trial and the numbers screened for eligibility, randomly assigned, offered and accepted interventions (eg, cohort multiple randomised controlled trials), received intended treatment, and analysed for the primary outcome.

#### Examples

**Fig 1:**
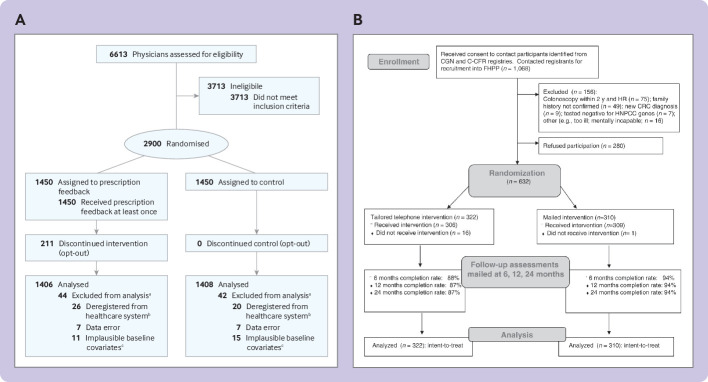
Examples of participant flowcharts for checklist item 13a of the CONSORT extension for randomised controlled trials conducted using cohorts and routinely collected data (CONSORT-ROUTINE).^60^ ^61^ (A) Adapted from Hemkens et al^21^ with permission. (B) Image reproduced from Lowery et al^63^ with permission

“We identified the primary care physicians with the highest antibiotic prescription rates in Switzerland using routinely collected claims data of prescriptions of antibiotics and outpatient consultations collected by SASIS, a data warehouse company of an umbrella organization of Swiss statutory health insurers (Santésuisse). These data are collected by over 60 statutory health insurers covering 64% of the Swiss population (5.1 million residents).We included among all board certified primary care physicians the 2900 top antibiotic prescribers (based on prescribed defined daily doses [DDD] per 100 consultations in the year prior to randomization…Of 2900 randomized physicians, all 1450 physicians in the intervention group received the evidence-based guidelines and first feedback information . . . Of the 1450 physicians, 211 (14.6%) opted out later. We used data from 2814 physicians for the intention-to-treat analysis”^21^ (fig 1A).

“Upon receiving permission to contact participants from their respective registry site, FHPP [Family Health Promotion Project] staff at the University of Colorado Cancer Center contacted participants to recruit them into the study (n=1,068). Of the 1,068 subjects contacted, 156 were deemed ineligible and 280 refused participation for an overall response rate of 69% (632 of 912 eligible . . .). The 632 consenting participants, representing 533 families, completed the baseline survey and were randomized to receive either the tailored telephone counseling intervention (N=322) or the general mailed intervention (N=310) . . . A total of 632 participants were enrolled in the FHPP trial. Of the 322 participants randomized to the telephone intervention, 306 (95%) received the intervention (16 participants could not be reached by phone within the allotted time frame per protocol), and 309 of 310 (>99%) participants in the mailed group received the mailed packet. Retention of participants over 24 months was greater than 90% overall: 87% in the telephone and 94% in the mailed intervention group”^63^ (fig 1B).

#### Explanation

The number of participants in a cohort or routinely collected database(s) and the numbers who were screened for eligibility, randomly assigned, offered and accepted interventions (eg, cohort multiple randomised controlled trials), received the intended treatment, and analysed for the primary and secondary outcomes should be described. When multiple sources of data were linked, potential exclusions because of data linkage should be specifically described. If people in a cohort or routinely collected database who are not included in the trial are observed and their data are reported, this should be clearly reported and included in the flowchart.

**Fig 2:**
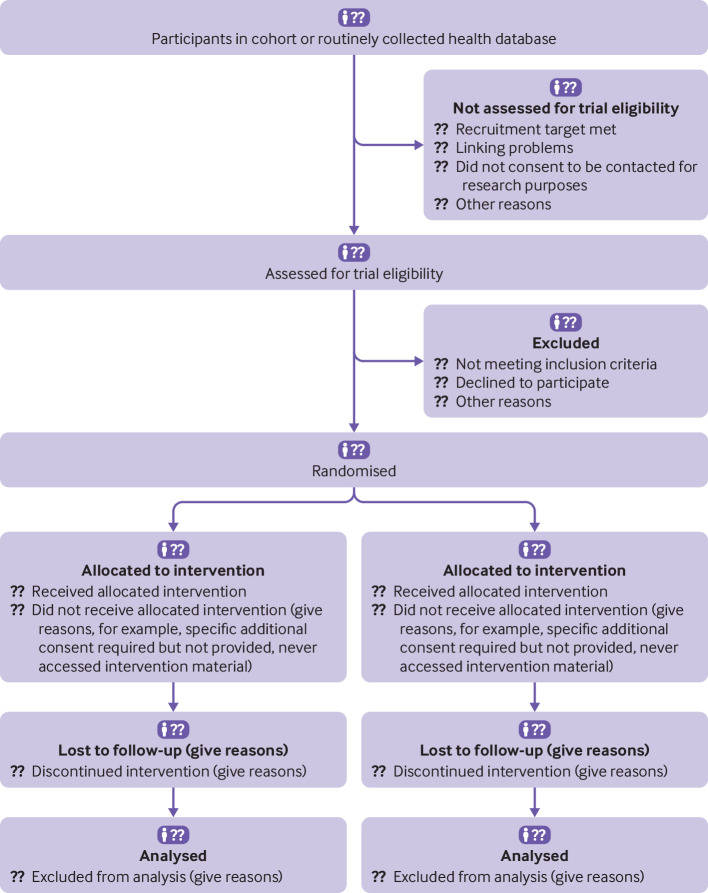
Example flow diagram for trials conducted using cohorts or routinely collected data

Figure 2 is an example of a flowchart that could be used to describe the flow of participants into a cohort or routinely collected database and then into the trial. Specific components to include depend on the trial design and might include the number of participants in the cohort or routinely collected database, the number who were not screened for eligibility for the trial because the recruitment target was met, data linkage problems were found, or participants did not consent to be contacted for research purposes, for example. Elements related to access or use of the intervention might also exist. For example, in the design for cohort multiple randomised controlled trials, consent for the intervention is sought after randomisation, in which case the number of participants who gave this consent should be reported.

#### Item 13b (unmodified)

For each group, losses and exclusions after randomisation, together with reasons (see CONSORT 2010).^8^ ^9^

Also, for trials using cohorts or routinely collected data, losses and exclusions based on data quality or linkage problems should be specifically described.

### Recruitment

#### Item 14a (unmodified)

Dates defining the periods of recruitment and follow-up.

#### Example

“A parallel group randomised controlled trial (RCT) with 878 participants in the intervention and 1,702 in the control group was performed between 2001-2002 . . . Briefly, 14 general practitioners with solo practices recruited participants for the RCT over a nine-month period starting in October 2000. Potential participants were identified using complete GP’s patient lists. At baseline (2000/2001), eligible study participants were at least 60 years old . . . Eligible individuals received the study information letter from their GPs, the PRA questionnaire (Probability for Repeated Admission) measuring six items of baseline risk status for health service use, i.e., person’s age, gender, hospital admissions, visits to GP, health status (heart disease and diabetes status), and caregiver availability, one question on B-ADL [basic activities of daily living] and the informed consent form.”^64^

#### Explanation

Participants in a cohort or routinely collected database are typically followed for an extended period, and the starting date of trial recruitment will often differ from the start date of data collection in the cohort or database. Trials with these types of data might be uniquely positioned to obtain long term follow-up data. The length of follow-up could be a fixed period after randomisation, but in randomised controlled trials when the outcome is time to an event, follow-up of all participants ends on a specific date. Start and end dates for the trial should be given, and the minimum, maximum, and median duration of follow-up for trials for which the outcome is time to an event should be reported. Longer term follow-up subsequent to a trial in an ongoing cohort or database, if expected, should be explained.

#### Item 14b (unmodified)

Why the trial ended or stopped (see CONSORT 2010).^8^ ^9^

### Baseline data

#### Item 15 (unmodified)

A table showing baseline demographic and clinical characteristics for each group.

#### Example

“During the study period, 11,709 patients with STEMI [ST-segment elevation myocardial infarction] in Sweden and Iceland underwent PCI [percutaneous coronary intervention] and were registered in SCAAR [Swedish Coronary Angiography and Angioplasty Registry]. Of these, 7012 were enrolled in the trial. An additional 247 patients were enrolled from the center in Denmark, for a total of 7259 patients…. Fifteen erroneous enrollments (patients initially reported as having STEMI, for whom the diagnosis was changed by the operator and no PCI was performed) were excluded from the database, leaving 7244 patients who underwent randomization. The baseline clinical characteristics of all the patients who underwent randomization (including patients at all the centers) and all the patients who did not undergo randomization (including patients at all the centers except the center in Denmark) are listed in Table 1.”^65^

#### Explanation

**Fig 3:**
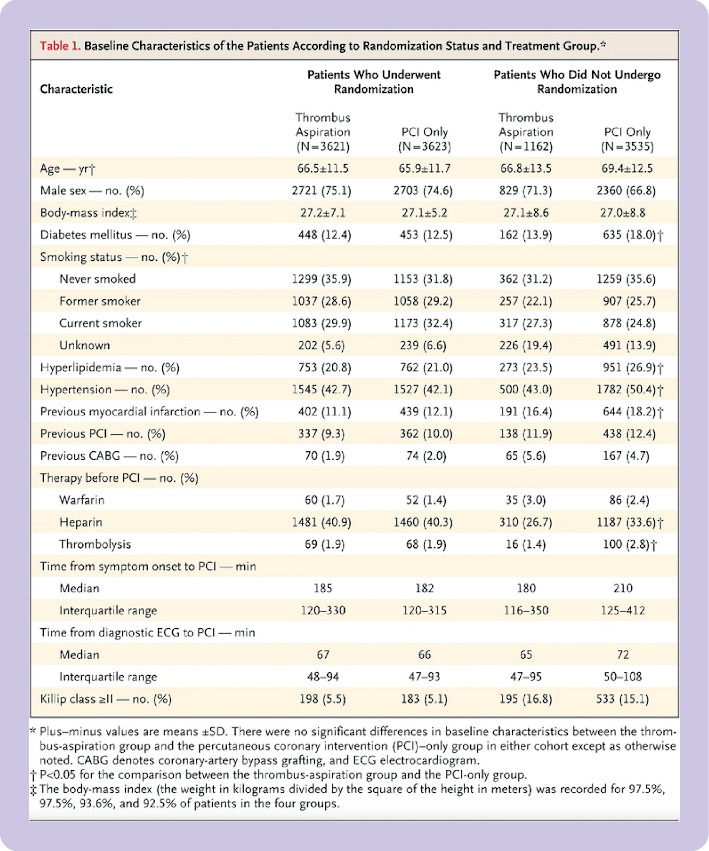
Example of table comparing baseline characteristics of participants in the trial and those who were not randomised for checklist item 15 of the CONSORT extension for randomised controlled trials conducted using cohorts and routinely collected data (CONSORT-ROUTINE).^63^ Image reproduced with permission

A feature of randomised controlled trials using cohorts and routinely collected data is that baseline data for participants not enrolled in the trial are usually more likely to be available. Figure 3 shows the table from the example above. Baseline characteristics for eligible people from the cohort or routinely collected database who were not eligible for the trial because of missing data or other administrative reasons, or who declined participation, should be reported in the same way, to the same extent as the randomised trial participants, if possible. Analyses that evaluate differences at trial entry between non-participants and those randomised can inform the representativeness of the participants in the trial.

### Numbers analysed

#### Item 16 (unmodified)

For each group, number of participants (denominator) included in each analysis and whether the analysis was by original assigned groups (see CONSORT 2010).^8^ ^9^

### Outcomes and estimation

#### Item 17a (unmodified)

For each primary and secondary outcome, results for each group, and the estimated effect size and its precision (such as 95% confidence interval) (see CONSORT 2010).^8^ ^9^

#### Item 17b (unmodified)

For binary outcomes, presentation of both absolute and relative effect sizes is recommended (see CONSORT 2010).^8^ ^9^

### Ancillary analyses

#### Item 18 (unmodified)

Results of any other analyses performed, including subgroup analyses and adjusted analyses, distinguishing pre-specified from exploratory (see CONSORT 2010).^8^ ^9^

### Harms

#### Item 19 (unmodified)

All important harms or unintended effects in each group (for specific guidance see CONSORT for harms; see CONSORT 2010).^8^ ^9^

## Discussion

### Limitations

#### Item 20 (unmodified)

Trial limitations, addressing sources of potential bias, imprecision, and, if relevant, multiplicity of analyses.

#### Examples

“A number of limitations of the TASTE [Thrombus Aspiration in ST-Elevation Myocardial Infarction in Scandinavia] trial should be noted. First, the treating physician was aware of the group to which the patient had been assigned, and that physician entered the angiographic variables into the registry; therefore, these variables were susceptible to bias. Second, we did not adjudicate events and did not review angiograms in a blinded fashion. We used all-cause death as the primary end point as it is the most stringent end point and because of the completeness of the national death registries in each participating country. We chose not to perform separate adjudication of secondary end points both to limit expense and because of the high reliability of the SWEDEHEART [Swedish Web System for Enhancement and Development of Evidence-Based Care in Heart Disease Evaluated According to Recommended Therapies] registry . . . A comparison of the clinical characteristics and outcomes between the patients who underwent randomization and those who did not indicates that the two cohorts differed significantly in a number of respects . . . Even when a trial uses a population-based registry for enrollment, the trial participants cannot be fully representative of the complete range of patients.”^65^

“Awareness of the trial might have itself promoted better data recording [in the EHR]. Nevertheless, we observed several limitations of the data including, for example, a high proportion of patients with unspecified subtype of stroke and a smaller number with BP [blood pressure] values not recorded during the intervention period. From an explanatory perspective, these limitations of the data reduce the capacity of the study to provide an accurate assessment of intervention efficacy.”^37^

#### Explanation

According to CONSORT 2010, identifying and discussing the potential limitations of a trial is crucial to appropriately interpretating the trial results, including issues such as potential bias, imprecision, and multiplicity of comparisons. Unique characteristics of trials using cohorts or routinely collected data might be linked to risk of bias and associated problems and, therefore, need specific attention in the discussion, including issues such as data availability, problems with data linkage, data validation, and data quality.^54^ The Clinical Trials Transformation Initiative^66^ has similarly identified that problems with the relevance, reliability, or reproducibility of data within registries or with other routinely collected data can influence the conduct and results of the trial.

### Generalisability

#### Item 21 (unmodified)

Generalisability (external validity, applicability) of the trial findings.

#### Example

“A comparison of the clinical characteristics and outcomes between the patients who underwent randomization and those who did not indicates that the two cohorts differed significantly in a number of respects, most notably in mortality at 30 days (2.9% among patients who underwent randomization vs. 10.6% among those who did not). In many cases, these differences reflect the exclusion from the trial of patients who were ineligible because they were unable to provide oral consent. Even when a trial uses a population-based registry for enrollment, the trial participants cannot be fully representative of the complete range of patients.”^65^

#### Explanation

Careful attention should be paid to how participants in an ongoing cohort or with records in a routinely collected database might differ from the population targeted by the trial, and these differences and their relevance for interpretating the findings of the trial should be discussed. Also, any trial design decisions related to delivery of the intervention or collection of outcomes that were influenced by the use of a cohort or routinely collected database should be considered. An advantage of many trials conducted in cohorts or with routinely collected data is that information on participants not included in the trial is available. Assessing the degree to which trial participants differ from non-participants by reason of non-participation can provide readers with insight on representativeness. Possible risks to generalisability that are identified, and their potential implications, should be discussed.

### Interpretation

#### Item 22 (modified)

CONSORT 2010 item: Interpretation consistent with results, balancing benefits and harms, and considering other relevant evidence.

Modified CONSORT extension item: Interpretation consistent with results, balancing benefits and harms, and considering other relevant evidence, including the implications of using data that were not collected to answer the specific research question.

#### Example

“Using the EHR [electronic health record] as a sole source of patient data is a limitation. For example, the EHR did not capture the patient experience of the intervention, including its potential impact on pain control, function, and disability. Furthermore, EHR data do not provide accurate substance use and mental health diagnoses. We did not have prescription or visit data from outside health systems.”^67^

#### Explanation

Authors should report whether and how the use of cohort or routinely collected data might be a limitation of the trial. These limitations could include, among others, the choice of outcome measures based on availability in the cohort or routinely collected database, and the quality and accuracy of the outcome data. Where possible, results should be compared with evidence from similar randomised controlled trials with a conventional design, and differences that might be related to the use of a cohort or routinely collected data should be discussed.

## Other information

### Registration

#### Item 23 (unmodified)

Registration number and name of trial registry.

### Protocol

#### Item 24 (unmodified)

Where the full trial protocol can be accessed, if available.

#### Example

“This trial used the platform of preexisting health care registries for enrollment, randomization, collection of data, and follow-up (for further details, see the Supplementary Appendix, available at NEJM.org).”^60^

#### Explanation

According to CONSORT 2010, trials should be registered, and their protocol should be accessible. When a trial is being conducted in a cohort or routinely collected database, in addition to the trial protocol, the authors should ideally provide a link to the protocol for the cohort or routinely collected database, if separate. This information allows interested readers to better understand the characteristics of the participants in the cohort or database and the data collection methods.

### Funding

#### Item 25 (modified)

CONSORT 2010 item: Sources of funding and other support (such as supply of drugs), role of funders.

Modified CONSORT extension item: Sources of funding and other support for both the trial and the cohort or routinely collected database(s), role of funders.

#### Example

“The registry is financed by the Swedish government and the Association of Local Authorities and Regions (the public health care provider), and is supported by the Swedish Heart Association, the National Board of Health and Welfare and the Swedish Heart and Lung Foundation. Participating hospitals are not reimbursed by the registry and costs of local data entry are borne by their internal budget [35, supplement]. The trial sponsor was the Karolinska Institutet.”^35^

#### Explanation

In addition to providing the funding source for the trial, authors should also report any funding sources of the cohort or routinely collected data, and if they were involved in the use of the cohort or dataset in the trial, or in the trial itself.

## Conclusions

This extension of the CONSORT reporting guideline for trials conducted using cohorts and routinely collected data are a minimum set of items to inform readers about the trial design and its findings, and to support informed decisions about the validity of the results of the trial and applicability to readers’ research questions. The extension only deals with aspects of trial reporting specific to trials conducted using cohorts and routinely collected data. When reporting a trial using cohorts or routinely collected data, authors should look at all items on the CONSORT checklist and use this document together with the main CONSORT 2010 guidelines. Authors should also consult other CONSORT extensions that are relevant to their trial design, such as extensions for cluster trials,^11^ pragmatic trial designs,^13^ or others. All are available online at www.consort-statement.org/extensions. Authors are also encouraged to report any extra information, specific to their trial, that would assist readers to more easily evaluate the results of the trial or to replicate the methods of the trial.

In addition to assisting authors of trial reports, this CONSORT extension aims to promote transparency and clarity, and to reduce research waste caused by poor reporting. We encourage journal editors to direct authors of trials conducted using cohorts and routinely collected data to use this checklist and to document adherence to reporting recommendations as a condition of manuscript submission.

## Web extra

Extra material supplied by authors

Web table: Combined CONSORT 2010 and CONSORT-ROUTINE Checklist

## Notes

Contributors: LK, OF, LGH, MZ, CR, SML, MSampson, DM, CG, EJ, and BDT were responsible for the study conception and design. MI, SJM, KAM, DBR, EIB, LT, MKC, DE, HMV, IB, PR, JN, RU, MSauvé, JF, and DT provided regular feedback on each step in the development process. LK, MI, SJM, OF, LGH, MZ, CR, DBR, SML, EIB, LT, MKC, DE, CG, EJ, and BDT contributed to drafting the manuscript. All authors provided a critical review and approved the final manuscript. BDT and LK are the guarantors. The corresponding author attests that all listed authors meet authorship criteria and that no others meeting the criteria have been omitted.

Data sharing: Additional data beyond that reported in the main and supplementary materials can be requested from the corresponding author.

Provenance and peer review: Not commissioned; externally peer reviewed.

Patient and public involvement: One of the members of the CONSORT extension for randomised controlled trials conducted using cohorts and routinely collected data (CONSORT-ROUTINE) team, MS, is a patient organisation leader. She was involved in working with researchers to establish a cohort of patients living with the rare disease scleroderma, which supports randomised controlled trials of online rehabilitation, self-management, and psychological intervention programmes.
